# High-brightness anterograde transneuronal HSV1 H129 tracer modified using a Trojan horse-like strategy

**DOI:** 10.1186/s13041-020-0544-2

**Published:** 2020-01-13

**Authors:** Peng Su, Min Ying, Zengpeng Han, Jinjin Xia, Sen Jin, Yingli Li, Huadong Wang, Fuqiang Xu

**Affiliations:** 1 Wuhan National Laboratory for Optoelectronics, Huazhong University of Science and Technology, Wuhan, 430074 China; 2 Center for Brain Science, State Key Laboratory of Magnetic Resonance and Atomic and Molecular Physics, Key Laboratory of Magnetic Resonance in Biological Systems, Wuhan Center for Magnetic Resonance, Wuhan Institute of Physics and Mathematics, Innovation Academy for Precision Measurement Science and Technology, Chinese Academy of Sciences, Wuhan, 430071 China; 3 University of Chinese Academy of Sciences, Beijing, 100049 China; 4 Huazhong University of Science and Technology (HUST)-Suzhou Institute for Brainsmatics, JITRI Institute for Brainsmatics, Suzhou, 215125 China; 5 Shenzhen Key Lab of Neuropsychiatric Modulation, Guangdong Provincial Key Laboratory of Brain Connectome and Behavior, CAS Key Laboratory of Brain Connectome and Manipulation, The Brain Cognition and Brain Disease Institute (BCBDI), Shenzhen Institutes of Advanced Technology, Chinese Academy of Sciences; Shenzhen-Hong Kong Institute of Brain Science-Shenzhen Fundamental Research Institutions, Shenzhen, 518055 China; 6 Center for Excellence in Brain Science and Intelligence Technology, Chinese Academy of Sciences, Shanghai, 200031 China

**Keywords:** Neural circuit tracing, Anterograde tracer, Herpes simplex virus, H129, Adeno-associated virus, Trojan horse-like strategy, High-brightness

## Abstract

Neurotropic viral transsynaptic tracing is an increasingly powerful technique for dissecting the structure and function of neural circuits. Herpes simplex virus type 1 strain H129 has been widely used as an anterograde tracer. However, HSV tracers still have several shortcomings, including high toxicity, low sensitivity and non-specific retrograde labeling. Here, we aimed to construct high-brightness HSV anterograde tracers by increasing the expression of exogenous genes carried by H129 viruses. Using a Trojan horse-like strategy, a HSV/AAV (adeno-associated virus) chimaera termed H8 was generated to enhance the expression of a fluorescent marker. In vitro and in vivo assays showed that the exogenous gene was efficiently replicated and amplified by the synergism of the HSV vector and introduced AAV replication system. H8 reporting fluorescence was brighter than that of currently available H129 tracers, and H8 could be used for fast and effective anterograde tracing without additional immunostaining. These results indicated that foreign gene expression in HSV tracers could be enhanced by integrating HSV with AAV replication system. This approach may be useful as a general enhanced expression strategy for HSV-based tracing tools or gene delivery vectors.

## Introduction

Transneuronal tracing of neural circuits using neurotropic viral tools is increasingly being recognized as a powerful approach for defining the synaptic organization of neural networks. Neurotropic viruses have the innate ability to invade neurons and produce infectious progeny that spread along synaptically connected neuronal networks [1–3]. Mapping neural circuits requires both anterograde and retrograde tracers [4]. To date, rabies virus and pseudorabies virus (PRV) strains have been modified for use as retrograde tracers [5–8]. Meanwhile, vesicular stomatitis virus (VSV) and herpes simplex virus type 1 (HSV-1) strain H129, originally isolated from the brain of a patient suffering from acute HSV encephalitis [9], have been reported to spread selectively in the anterograde direction and are widely used as anterograde tracers [10–12]. However, HSV H129 tracers still have several drawbacks, such as high toxicity, low sensitivity, and non-specific labeling. With current trans-multisynaptic H129 tracer systems, sampling takes place 3 days after intracranial injection because experimental animals die within 3–5 days of infection [13, 14]. Because the fluorescent protein gene carried by H129 tracers is not highly expressed in 3 days, it is usually necessary to amplify the fluorescent signal by immunohistochemistry to visualize and dissect the structure of the output neural networks [15–17]. For instance, immunostaining for GFP protein provides sensitive detection and high contrast for image analysis. Therefore, a H129 tracer with increased brightness would be more convenient for anterograde transsynaptic tracing. Furthermore, decreasing the sampling time could increase the likelihood that labeled brain regions are directly related to the injection site. Thus, enhancing the expression of exogenous genes is one of the core objectives for improving HSV1 H129-based viral tracers.

Here, we aimed to increase the expression of exogenous genes carried by H129 tracers by constructing a HSV/adeno-associated virus (AAV) chimaera virus. AAVs are naturally replication-defective human parvoviruses. With their small and relatively simple genomes, AAVs have become preferred viral vectors for gene delivery, primarily because they exhibit little or no toxicity and do not cause inflammation [18–20]. AAVs require the presence of a helper virus (adenovirus or HSV) to trigger replication and the generation of progeny viruses [21, 22]. As such, one of the most important large-scale production methods for the generation of recombinant AAV (rAAV) vectors is employing the HSV platform [23, 24]. Furthermore, AAVs can sometimes assist in enhancing the expression of transgenes in plasmid vectors. For example, plasmid-based HSV pseudoviral amplicon vectors show great promise in gene therapy and have been studied for several decades [25, 26]. Studies have shown that long-term transgene expression can be achieved when the AAV *rep* gene is added along with the transgene of interest into the HSV-1 amplicon plasmid, with the transgene flanked with AAV inverted terminal repeat elements (*ITRs*) [27–29].

**Fig. 1:**
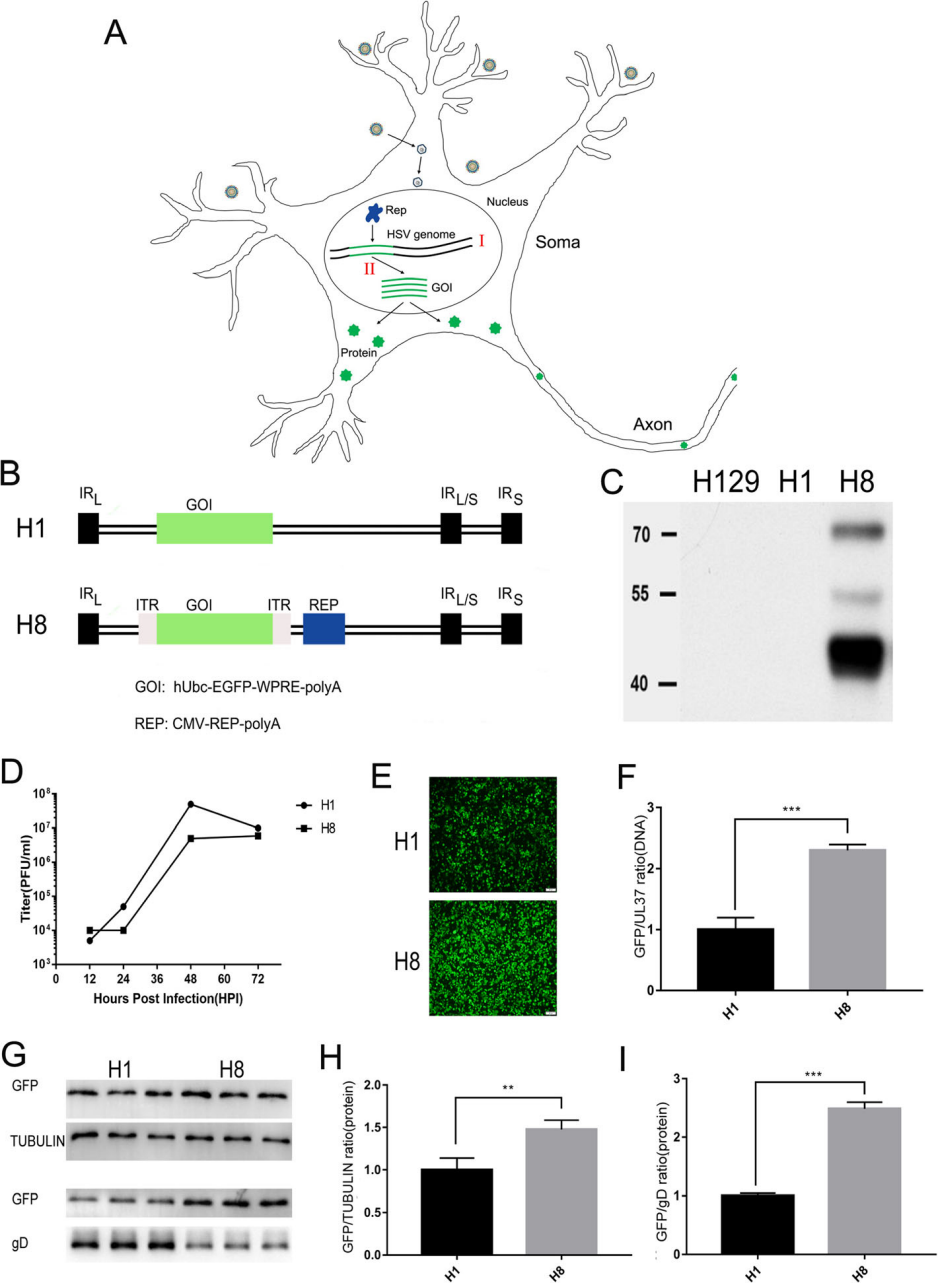
Construction and validation of H1 and H8 viruses in vitro. **a** The Trojan horse-like strategy of GOI amplification by HSV/AAV chimaera. AAV replicase expression cassette and *ITR* flanked GOI (gene of interest) cassette were inserted into H129 genome to construct the chimaera virus. The genome of the HSV chimaera enters the nucleus following infection of cells. With the assistance of HSV, the Rep replicase specifically recognizes GOI flanked by *ITRs* and replicates it, resulting in amplification of the copy numbers of the GOI. **b** Schematic of H1 and H8 genome. IRL and IRS indicate long and short inverted repeats, respectively. **c** Western blot validation of rep protein expression. H129, H1 and H8 infected BHK cells were detected using the anti-replicase antibody. **d** Single-step growth curves for H1 and H8 (each point represents the mean of triplicate assays). **e** Fluorescent protein expression level of H1 and H8 infected BHK cells at 24 h post infection, MOI = 5. Scale bar, 100 μm. **f** H8 showed significant higher DNA level of *egfp* than H1. **g**, **h**, **i** H8 showed significant higher protein level of GFP than H1. The genomes and proteins of H1 and H8 infected BHK cells were extracted 48 hpi

In the present study, we designed and applied a Trojan horse-like strategy whereby an AAV replication system was directly introduced into the HSV viral genome to enhance the replication and expression of an enhanced green fluorescent protein gene (*egfp*), which is different from the plasmid-based pseudoviral hybrid amplicon system (Fig. 1a). We constructed H1 (routine tracer) and H8 chimeric viruses (novel high-brightness tracer) containing identical *egfp* expression cassettes except that H8 also contained an AAV replication system to control the replication and expression of the *egfp* gene. In vitro and in vivo assays showed high levels of exogenous gene expression by the H8 virus, suggesting that the chimeric virus could be more convenient and flexible for anterograde transneuronal tracing.

## Materials and methods

### Animals

All experimental and surgical procedures were conducted in accordance with the guidelines of the Animal Care and Use Committees at the Wuhan Institute of Physics and Mathematics, Chinese Academy of Sciences. Adult male C57BL/6 mice were purchased from Hunan SJA Laboratory Animal Company. All animals were housed in a dedicated housing room with a 12/12 h light/dark cycle, and food and water were available ad libitum. All the experiments with viruses were performed in bio-safety level 2 (BSL-2) laboratory and animal facilities.

### Cells and viruses

The wildtype HSV-1 H129 strain was generously provided by Professor Lynn Enquist (Princeton University, Princeton, NJ). Viral stocks were grown on Vero cells maintained in Dulbecco’s minimum essential media (DMEM) with 10% fetal bovine serum (FBS). Standard plaque assays were performed by serially diluting virus in DMEM supplemented with 2% FBS (2% DMEM) and overlaying infected cells with medium containing 2% FBS, antibiotics, and 1% agarose. Plaques were identified using neutral red staining and/or fluorescence microscopy where appropriate. Aliquots of viral stocks were stored frozen at − 80 °C.

### Construction of H129 recombinant H1 and H8 viruses

To construct H1, the EGFP expression cassette was inserted into the intergenic region between the *UL37* and *UL38* genes. The *UL37* and *UL38* homology arms and reporter gene were cloned into the vector pcDNA3.1+ to generate the targeting plasmid. Briefly, *UL37-UHA* (upstream homology arm) and *UL38-DHA* (downstream homology arm) were generated by PCR using H129 genome DNA as the templates. Subsequently, the homology arms were ligated into the pcDNA3.1+ vector and a new MCS site (HpaI-AgeI-BamHI-SbfI) was inserted between the two arms. The EGFP expression cassette containing hUbC promoter, *EGFP* gene, *WPRE* and *bGH* ploy (A) signal was inserted between *UL37* and *UL38* homology arms to create *pH 129-hUbC-EGFP-WPRE*.

The strategy adopted to produce H8 virus was to introduce AAV replication system into HSV genome to construct HSV/AAV chimeric virus. AAV replicase expression cassette (*CMV-Rep-PA*) and *ITR* as flanking elements of fluorescent gene expression cassette were also inserted into the intergenic region between the *UL37* and *UL38* genes (Fig. 1b). In brief, the codon-modified *Rep* gene of AAV serotype 2 expression cassette (*CMV-Rep-PA*) was digested with SbfI and AgeI, and then introduced into the same enzymes digested 3.1 + 37 arm-38 arm vector to create *pH 129-CMV-Rep* plasmid. The EGFP expression cassette containing *hUbC* promoter, *EGFP* gene, *WPRE* and *bGH* ploy (A) signal was inserted between the two *ITRs* of the cloning vector *pFD-(ITR-GFP)*. Then the whole *ITR* flanked *EGFP* expression cassette was digested with SbfI and ligated into SbfI-digested *pH 129-CMV-Rep* vector to create *pH 129-CMV-Rep/ITR-hUbC-EGFP-WPRE-ITR* targeting vector. Successful construction and function of the targeting plasmid was confirmed by restriction enzyme digestion, sequencing and transfection into 293 T cells (data not shown).

To generate H1 (*rH129-hUbC-EGFP-WPRE*) and H8 (*rH129-CMV-Rep/ITR-hUbC-EGFP-WPRE-ITR*) recombinant viruses, the corresponding expression cassettes were inserted the H129 genome via homologous recombination. The resulting plasmids *pH 129-hUbC-EGFP-WPRE* and *pH 129-CMV-Rep/ITR-hUbC-EGFP-WPRE-ITR* were purified by Omega plasmid mini kit and separately co-transfected with H129 genomic DNA into 293 T cells in six-well plates, using Lipofectamine 2000 (Invitrogen) according to the manufacturer’s instructions. After the majority of cells showed cytopathic effect, the medium was removed and the cells were harvested in PBS. After three-rounds of freeze-thaw-vortex, the cell lysate was used to infect Vero cells plated in six-well plates. After one hour of infection, viruses were removed and DMEM with 2% FBS, antibiotics, and 1% agarose were overlaid on the cells. After 2 or 3 days, well-separated EGFP-expressing plaques were picked and subjected to at least five more rounds of plaque-purification to remove wild type H129 virus.

The novel purified H1 and H8 viruses were mass-produced by infecting Vero cells grown in T75 tissue culture flasks. After infected cells showed a prominent cytopathic effect (~ 2 days), medium containing the viruses was collected, centrifuged to remove cell debris (7000 g for 10 min), the supernatant passed through a 0.22 μm filter, and finally centrifuged at 50,000 g/3 h using Beckman Avanti J-26SXP Ultracentrifuge. The virus pellet was resuspended overnight at 4 °C in a small amount of cold PBS. Dissolved viruses were aliquoted into 3 μl and stored at − 80 °C. The titer of viral stocks was determined using standard plaque assay on Vero cells and titers were expressed as plaque-forming units (PFU) per milliliter. A fresh aliquot of stock virus was thawed and used for each experiment. The titers of virus stocks used in these studies were ~ 3 × 10^9^ PFU/ml for H1 or H8 virus.

### Viral growth kinetics analysis

BHK-21 cells (5 × 10^5^) were plated into six-well plates, and infected with either H1 or H8 at a multiplicity of 10 PFU/cell. After 1 h of adsorption, the viral inocula were removed (time point 0), cells were washed 3 times with PBS to remove unabsorbed virus and DMEM medium (containing 2% FBS) was added. Supernatants were collected at different time points as indicated (12, 24, 48 and 72 h post infection (HPI)) and virus titers were determined by standard plaque assays.

### Q-PCR and Western blot

#### Q-PCR assay

For in vitro test, the BHK cells were infected with H1 or H8 virus at MOI = 0.1 in a six-well plate, respectively. After 48 h, the supernatant was discarded and the cells were rinsed with PBS before scraped down for DNA or protein extraction. For in vivo detection, 200 nl H1 or H8 virus was injected into midbrain VTA region of 4 adult C57BL/6 mice, respectively. Three days after injection, mice brains were collected and ground evenly in liquid nitrogen with 1 ml lysis buffer. Then, 100 μl lysate of each brain was used to extract genome or protein. The genome was extracted using the Tissue DNA Kit (D3396, OMEGA). For protein extraction, cells or brain tissues were resuspended in cell lysis buffer (Beyotime Biotechnology), stand at room temperature for 10 minutes, centrifuged at 10000 g for 10 minutes and then remain the supernatant.

Gene copy number were determined by qPCR assay with the iQ SYBR Green Supermix kit (Bio-Rad). A standard curve was obtained using 10-fold serial dilutions of plasmid *pH 129-CMV-Rep/ITR-hUbC-EGFP-WPRE-ITR*. The quantitative primers for *UL37* and *eGFP* were *Q-UL37-F* (ACAGCGTAGACCAACGACGAGA) and *Q-UL37-R* (AACGGGATGCCGGGACTTA), *Q-eGFP-F* (AAGCAGAAGAACGGCATCA) and *Q-eGFP-R* (GGCGGTCACGAACTCCA), respectively.

#### Western blot

Samples were denatured in 5× loading buffer at 99 °C for 8 min, and separated by using 10% SDS-PAGE electrophoresis and then transferred onto polyvinylidene fluoride (PVDF) membranes. GFP proteins were detected using a rabbit anti-GFP antibody (ab290, Abcam) at a 1:2000 dilution. HSV gD proteins were detected using a mouse monoclonal antibody (ab6507, Abcam) at a 1:5000 dilution. A monoclonal antibody against β-tubulin (Proteintech) was used at a 1:8000 dilution. Secondary detection antibodies were used at a 1:5000 dilution. The images were collected and analyzed by gel-imaging (BioRad, ChemiDocTM MP).

### Stereotactic surgery

All procedures on animals were performed in Biosafety level 2 (BSL-2) animal facilities as before [30]. Animals were anesthetized with pentobarbital sodium by intraperitoneal injection (80 mg/kg, i.p.), and placed in a stereotaxic apparatus (Item: 68030, RWD, Shenzhen, China). During surgery and virus injection, all animals were kept anesthetized with isoflurane (1–1.5%). The skull above the targeted areas was thinned with a dental drill and removed carefully. Injections were conducted with a syringe pump (Item: 53311, Quintessential stereotaxic injector, Stoelting, United States) connected to a glass micropipette with a tip diameter of 10–15 mm. The glass micropipette was held for an extra 10 min after the completion of the injection and then slowly retreated. After the surgery, the incisions were stitched and lincomycin hydrochloride and lidocaine hydrochloride gel was applied to prevent inflammation and alleviate pain for the animals.

6 × 10^5^ PFU H1 or H8 was injected into the adult male C57BL/6 mice (*n* = 3) with the following coordinates: VTA (AP, − 3.20 mm; ML, − 0.40 mm; and DV, − 4.30 mm), V1 (AP, − 2.80 mm; ML, − 2.40 mm; and DV, − 0.90 mm), NAc (AP, + 1.0 mm; ML, − 1.1 mm; and DV, − 4.50 mm). To determine whether H8 might allow neural circuit labeling in a shorter time period, H8 was injected into the accumbens nucleus (NAc) of adult C57BL/6 mice and collected samples at 24, 36, and 48 h post-injection.

### Slice preparation and confocal imaging

The mice were anesthetized with pentobarbital sodium (100 mg/kg body weight, i.p.), and perfused transcardially with PBS (5 min), followed by ice-cold 4% paraformaldehyde (PFA, 158127 MSDS, sigma) dissolved in PBS (5 min). The brain tissues were carefully removed and post-fixed in PBS containing 4% PFA at 4 °C overnight, and then equilibrated in PBS containing 25% sucrose at 4 °C for 72 h. The 40 μm thick coronal slices of the whole brain were obtained using the cryostat microtome and stored at − 20 °C.

For all samples, every sixth section of the brain slices were selected, stained with DAPI, washed with PBS, mounted with 70% glycerol (in PBS) and sealed with nail polish. All of the images were captured with the Olympus VS120 virtual microscopy slide scanning system (Olympus, Shanghai, China).

### Data analysis

SPSS (version 13.0) and Origin 9.0 were used for data analysis (student’s *t*-tests) and statistical graphs, respectively. All data were presented as means ± SEM. Statistical significance was set as ^*^P < 0.05, ^**^P < 0.01 and ^***^P < 0.001.

## Results

### Construction and characterization of the H8 chimeric virus in vitro

The H8 chimeric virus was generated by the introduction of the AAV replication system (replicase and *ITR* elements) into the HSV1 strain H129 genome to enhance the expression of an exogenous gene of interest (GOI), which, in this case, was *egfp*. Figure 1a showed the construction strategy used in this study. While the H1 and H8 viruses contained the same *egfp* expression cassettes, H8 also contained the AAV replication system to enhance the replication of the *egfp* gene (Fig. 1b). Western blotting experiments showed that the H8 virus effectively expressed the AAV Rep protein, while no Rep expression was detected in H1- or wild type H129-infected cells (Fig. 1c). Analysis of viral growth kinetics showed that H8 replication was only slightly slower than that of H1, which can be observed in the single-step replication curve (Fig. 1d).

As expected, the green fluorescence of H8 chimeric virus-infected cells was significantly brighter than that of H1-infected cells (Fig. 1e). qPCR analysis was conducted to quantify the *egfp* expression cassette copy number, while the amplified *UL37* gene fragment was selected as an indicator of the HSV genome copy number. One HSV genome has only 1 copy of the *UL37* gene, which was consistent with the qPCR result of *egfp*/*UL37* ratio of H1 (Table 1). The *egfp*/*UL37* ratio from H1 was defined as 1. The results showed that the *egfp*/*UL37* ratio for the H8 virus was significantly higher than that of the H1 virus (Table 1 and Fig. 1f), indicating that there was a greater number of copies of *egfp* in the H8-infected cells than in the H1-infected cells. Thus, these findings confirmed that the Rep protein expressed by the HSV/AAV chimeric vector could effectively replicate the *ITR*-flanked exogenous gene. The *egfp*/*UL37* ratio for H8 was ~ 2.30 (± 0.06), indicating that its *egfp* copy number was > 2-fold higher than that of the H129 genome.

**Table 1:** Q-PCR assay was conducted to quantify the *egfp* expression cassette copy number in H1 or H8 infected cells *in vitro*^a^

Sample	*UL37* quantity	*UL37* adjusted quantity	*egfp* quantity	Adjusted relative ratio	Average ratio
H1	21,996,416.00	23,379,909.17	18,561,812.00	0.79	1.00 ± 0.11
H1	21,587,338.00	22,945,101.68	23,500,664.00	1.02	
H1	20,390,316.00	21,672,791.42	25,614,332.00	1.18	
H8	14,824,173.00	15,756,558.63	37,436,844.00	2.38	2.30 ± 0.06^b^
H8	13,998,001.00	14,878,423.46	34,695,536.00	2.33	
H8	14,782,463.00	15,712,225.22	34,447,996.00	2.19	

^a^ BHK cells were infected with H1 or H8 virus at MOI = 0.1 in a six-well plate. After 48 h, the supernatant was discarded and the cells were rinsed with PBS before scraped down for DNA or protein extraction. Gene copy number of GOI were determined by qPCR assay with the iQ SYBR Green Supermix kit (Bio-Rad). Results were expressed as means ± SEM of each group

^b^ Significant difference (*p* < 0.001), compared with H1 group

Western blotting was then performed to quantify GFP expression by both H1 and H8. To exclude the effect of amplification speed differences, both cellular tubulin and HSV glycoprotein D were selected as internal references (Fig. 1g). For quantification, the EGFP/IR (internal reference) ratio from the H1 virus was defined as 1. When tubulin was used as the internal reference, the EGFP/IR ratio for strain H8 was 1.48 (± 0.06) (Additional file 1: Table S1), whereas a ratio of 2.49 (± 0.07) (Additional file 1: Table S2) was recorded when glycoprotein D was used as the internal reference (Fig. 1h, i). Taken together, these results showed that EGFP copy number and expression was significantly higher in H8-infected cells than in H1-infected cells.

### In vivo validation of GOI expression enhancement of the H8 chimeric virus

**Fig. 2:**
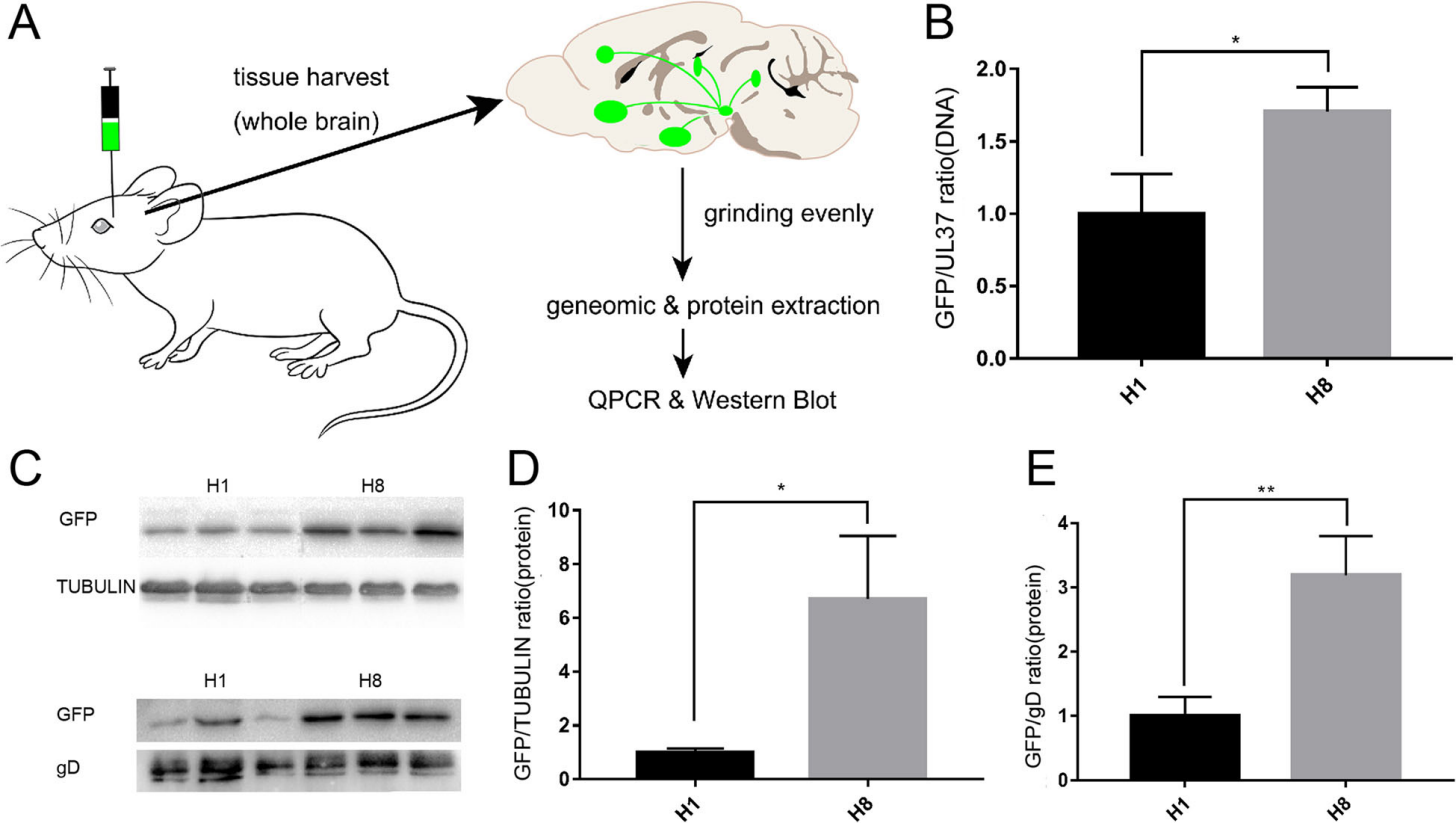
Validation of high fluorescence intensity of H8 in vivo. **a** The schematic diagram of virus injection and sample treatment. H1 or H8 virus was injected into midbrain VTA region of mice, respectively. All brains were taken 72 hpi and ground evenly in liquid nitrogen and then 100 μl lysate of each brain was used to extract genome or protein. **b**-**e** H8 showed significant higher EGFP DNA copies and protein levels than that of H1 in vivo

To test whether H8 produced a strong labeling signal in vivo, we separately injected H1 and H8 into the VTA of adult C57BL/6 mice. At 3 days post-injection, whole brains were harvested from infected mice and used for qPCR and Western blotting analyses to determine *egfp* gene and protein levels (Fig. 2a).

Not unexpectedly, in vivo experiments showed higher levels of *egfp* in H8-infected brains compared with H1-infected brains (Fig. 2b). At the gene level, the *egfp*/*UL37* ratio for H8 was ~ 1.71 (± 0.10), indicating that the *egfp* copy number was 1.7-fold higher than the HSV genome copy number (Table 2). At the protein level, a correspondingly higher EGFP expression level was observed in H8-infected mice compared with H1-infected mice (Fig. 2c). When tubulin was used as the internal reference, the EGFP/IR ratio for H8 was 6.71 (± 1.36) (Additional file 1: Table S3), and a ratio of 3.19 (± 0.35) (Additional file 1: Table S4) was determined when using glycoprotein D as the internal reference (Fig. 2d, e). The results indicated that GOI copy number and protein expression was significantly higher in H8-infected mice compared with H1-infected mice.

**Table 2:** In vivo gene copy number of exogenous gene in H1 or H8 infected mice brains were assessed using Q-PCR assay^a^

Sample	*UL37* quantity	*UL37* adjusted quantity	*egfp* quantity	Adjusted relative ratio	Average ratio
H1	6,028,302.00	9,995,136.49	7,109,552.50	0.71	1.00 ± 0.16
H1	16,667,208.00	27,634,816.37	28,367,098.00	1.03	
H1	2,208,835.25	3,662,326.44	4,622,589.50	1.26	
H8	5,432,914.50	9,007,963.10	13,658,822.00	1.52	1.71 ± 0.10^b^
H8	5,167,673.00	8,568,183.37	15,538,736.00	1.81	
H8	7,823,168.50	12,971,088.20	23,258,302.00	1.79	

^a^ For in vivo detection, 200 nl H1 and H8 virus was injected into midbrain VTA region of C57BL/6 mice, respectively. Three days after injection, mice brains were collected and ground evenly in liquid nitrogen with 1 ml lysis buffer. Then, 100 μl lysate of each brain was used to extract genome or protein. Gene copy number of GOI were determined by qPCR assay with the iQ SYBR Green Supermix kit (Bio-Rad). Results were expressed as means ± SEM of each group

^b^ Significant difference (*p* < 0.05), compared with H1 group

Comparison of neural circuit tracing efficiency of H8 and H1 in vivo was conducted. H8 and H1 were separately injected into the VTA of C57BL/6 mice. Whole brains were harvested from infected mice 3 days later. Brain slices confirmed that EGFP expression by the H8 virus was obviously higher than that of H1, with differences even apparent to the naked eye (Additional file 1: Figure S1A). H8 viruses could efficiently label the neurons with high fluorescent intensity following intracerebral injection. Many neurons of both injection site and various VTA output brain regions were well labeled. The fluorescence intensity of H8 was sufficient to clearly label and visualize the neuronal cell bodies and nerve fibers (Additional file 1: Figure S1B). These results confirmed that the HSV/AAV chimeric viral tracers worked well in vivo, and H8 traced output neural circuits more efficiently, and fluorescence labeling was much brighter than that of H1. Meanwhile, it was notable that H8 might exhibit some non-specific retrograde labeling. Our previous research found that H129 derived tracers were also able to infect neurons of upstream brain regions via direct retrograde uptake/transmission, especially at longer post-inoculation intervals [31]. The labeled hippocampus or dorsal raphe by H8 might be the direct retrograde labeling regions from the VTA injection site.

### In vivo validation of the anterograde transport phenotype of H8 virus as wildtype H129

Recombinant H1 constructed based on wild type H129 was used to be an anterograde tracer, and it has been used to dissect output neural circuits in many cases. Since H8 was constructed at the same locus of wild type H129 as H1, we suspected that H8 would possess the same transport phenotype as H1.

**Fig. 3:**
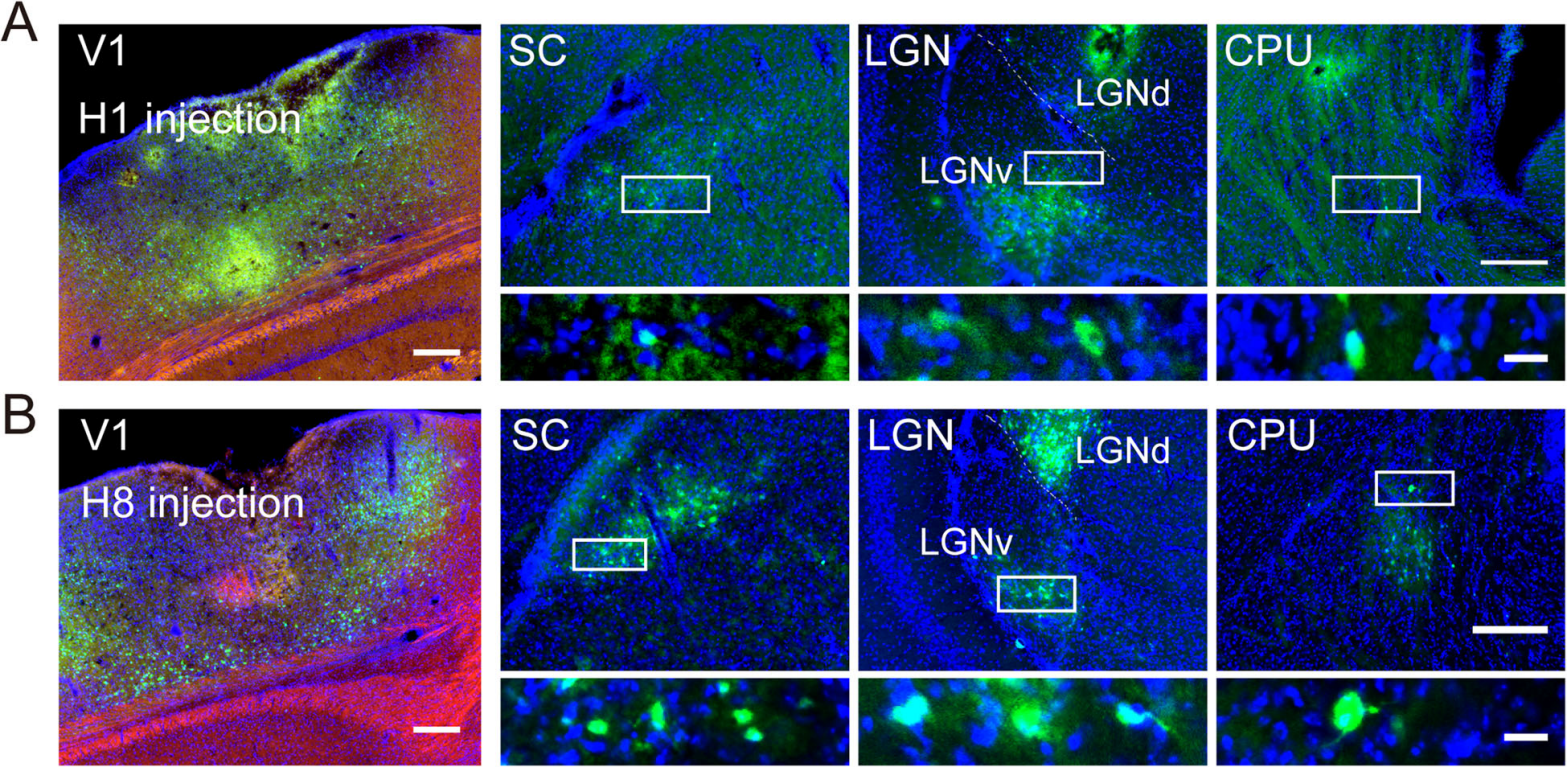
Validation of anterograde transport phenotype of H8 in vivo. **a** Left, GFP expression in the injection site following injection of H1 into V1 of C57BL/6 mice. Blue was DAPI staining. Right three panels, GFP fluorescence in the regions downstream of V1 (SC, LGN, and CPU). High-magnification images (bottom) showed GFP-labeled cell bodies in the region indicated by the white box. **b** Left, GFP expression in the injection site following injection of H8 into V1 region of mice. Right three panels, GFP fluorescence in the regions downstream of V1 (SC, LGN, and CPU). High-magnification images (bottom) showed GFP-labeled cell bodies in the region indicated by the white box. Scale bars, 200 μm, left and right top panels; 20 μm, right bottom panels

To validate this suspicion, V1 (primary visual cortex) injection of H1 and H8 experiments was performed. Three days after injection, the mouse brains were collected for imaging. We observed GFP-expressing cell bodies in regions known to be directly projected by V1, including the superior colliculus (SC), ventral lateral geniculate nucleus (LGNv), and caudate putamen (CPU) (Fig. 3). As SC and CPU supposedly should not project back to V1 [32, 33], the presence of GFP^+^ cell bodies in these areas may be explained by the anterogradely transneuronal spread of the virus from V1 axons to neurons in their targeted structures. In the brain regions that were reciprocally connected with V1 (e.g., dorsal lateral geniculate nucleus, LGNd), GFP labeling of cell bodies might result from both anterograde and retrograde transport of the virus. These regions were not considered in the current study because of the ambiguity. Due to the observation of the same labeling profiles for H1 (Fig. 3a) and H8 (Fig. 3b) in vivo, the results confirmed that H8 could also be used as an anterograde tracer.

### Using H8 to dissect neural circuits in a shorter timeframe

**Fig. 4:**
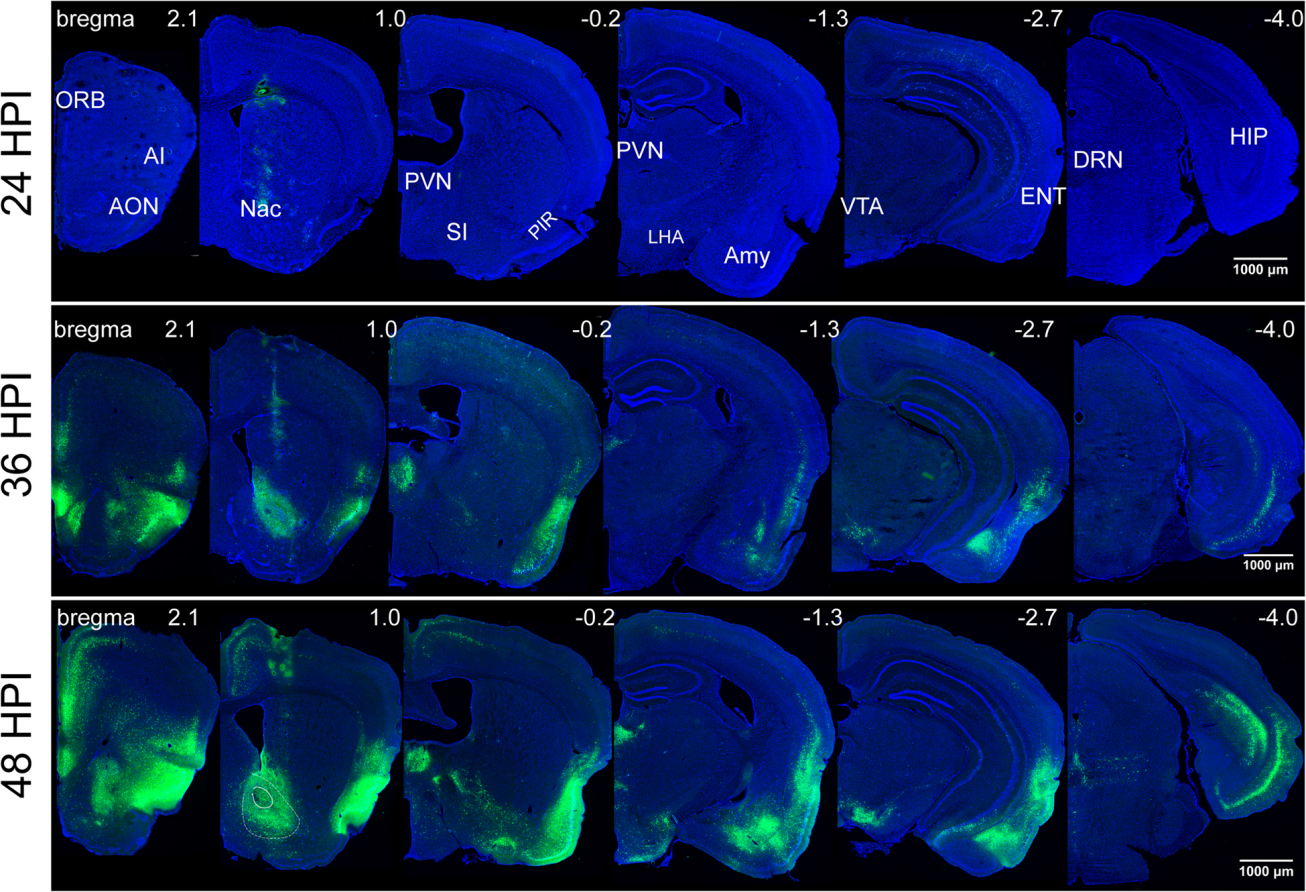
Labeling efficiency of H8 in tracing NAC output pathway at different timeframe. Three early infection stages were chose to show the labeling performance of H8. H8 was injected into the accumbens nucleus (NAc) of adult C57BL/6 mice and collected samples at 24, 36, and 48 h post-injection. Scale bar, 1000 μm. anterior olfactory nucleus (AON), orbital cortex (ORB), agranular insular cortex (AI), piriform cortex (Pir), substantia innominata (SI), paraventricular thalamic nucleus (PVN), amygdala (Amy), entorhinal cortex (ENT), hippocampus (HIP), ventral tegmental area (VTA), lateral hypothalamus (LHA), dorsal raphe nuclei (DRN)

**Fig. 5:**
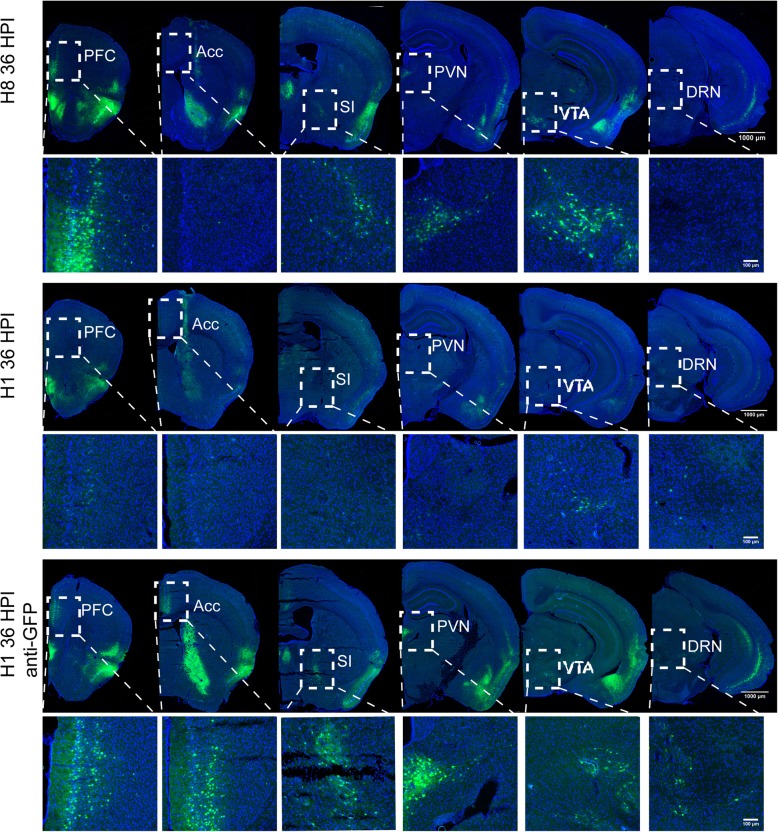
Comparison of labeling performance of H8 and H1 at 36 h post infection. H8 and H1 were injected into the accumbens nucleus (NAc) of C57BL/6 mice respectively and the brain samples were collected at 36 h post-injection. Serial coronary slices were displayed to show the differences between H8 and H1 (Scale bar =1000 μm) and smaller areas in dashed boxes were magnified (Scale bar = 100 μm). prefrontal cortex (PFC), anterior cingulate cortex (Acc), substantia innominata (SI), paraventricular thalamic nucleus (PVN), ventral tegmental area (VTA), dorsal raphe nuclei (DRN)

The observed higher fluorescent protein expression for the H8 virus might allow neural circuit labeling in a shorter time period. To examine this possibility, we injected H8 into the nucleus accumbens (NAc) of adult C57BL/6 mice and collected samples at 24, 36, and 48 h post-injection (Fig. 4 and Fig. 5).

**Fig. 6:**
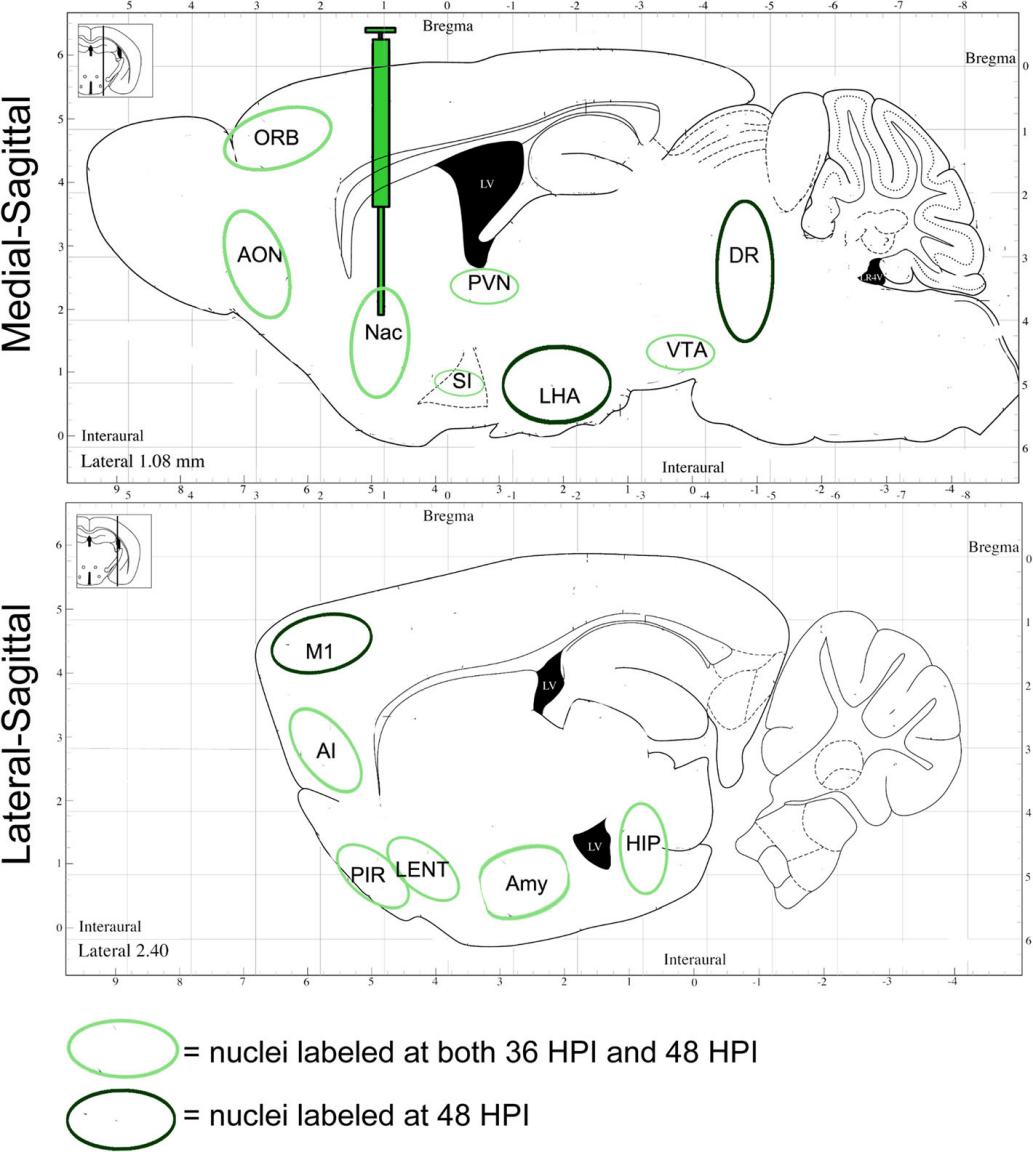
The structural neural networks labeled by H8 in the NAc output pathway. Medial-sagittal and lateral-sagittal plates and labeled structures are from the Franklin and Paxinos Atlas (Franklin and Paxinos, 2001). Light green circles mark GFP-labeled structures by H8 tracer at both 36 hpi and 48 hpi; dark green circles mark GFP-labeled structures at 48 hpi

At 24 h post-injection, fluorescence was only observed at the injection site, indicating no transneuronal spread of the H8 virus. However, at 36 h post-injection, fluorescence was observed in many discrete brain areas, including the anterior olfactory nucleus (AON), the orbital cortex (ORB), the agranular insular cortex (AI), the piriform cortex (Pir), the substantia innominata (SI), the paraventricular thalamic nucleus (PVN), the amygdala (AMY), the entorhinal cortex (ENT), the hippocampus (HIP), and the ventral tegmental area (VTA), amongst others. Nuclei labeled at this time point may be directly connected to or receive the strongest outputs from the accumbens nucleus [34]. After a longer infection time (48 h post-injection), fluorescence was detected across a much wider range of brain regions, including the lateral hypothalamus (LHA), dorsal raphe nuclei (DRN), and most parts of the cerebral cortex (Fig. 4). Thus, these regions might be indirect neural outputs from the injection site (Fig. 6). It was speculated that H8 injected into the nucleus accumbens might be transmitted anterogradely across multiple synapses to infect and label the cerebral cortex.

As a control, we also examined H1 EGFP expression at 36 h post-injection. At this time point, all brain regions labeled by H8 were also traced by H1, although GFP intensity was much lower than that of H8. However, after immunohistochemical staining using anti-GFP antibody, the H1 virus labeled a greater number of brain regions than H8 (Fig. 5). These findings indicated that H8 could be used as a more bright and flexible anterograde neural circuit tracer without immunostaining, compared with the currently available HSV tracers.

## Discussion

To fully understand information transfer throughout the nervous system, the connections among neurons need to be defined first. This requires careful definition of the synaptic inputs and outputs of specific neuronal subpopulations in different regions. HSV1 strain H129 is the most widely used anterograde transsynaptic tracer and has a relatively high transneuronal spread efficiency. The expression of fluorescent protein by H129 makes it simple to dissect neural circuits using an optical microscope. As such, higher fluorescein expression levels make it easier to detect the viral tracer labeling. However, the majority of currently available H129 viral tracers are limited by their low gene expression levels. Therefore, it is usually necessary to amplify the fluorescent signal by immunostaining using fluorescein-specific antibodies to clearly visualize labeling signals. To address the problem of low sensitivity, several different approaches have been used to enhance fluorescent protein expression in H129 tracers, including using strong promoters, expression regulators, or the insertion of several copies of fluorescent protein-encoding genes into the viral genome. However, these methods only add a limited number of copies of the transgene because HSV genomes undergo homologous recombination of repeated gene sequences. Thus, the transgene copy number is limited by HSV itself, restricting fluorescent protein expression.

Here, we designed and applied a Trojan horse-like strategy in order to construct a chimeric HSV/AAV vector to overcome the limitation of low fluorescent protein expression of H129-based vectors. We developed a chimeric virus, named H8, with high levels of fluorescent protein expression both in vitro and in vivo. The strategy was based on two important premises. First, AAV genomes have a simple and efficient replication system that requires the assistance of a helper virus (adenovirus or HSV) to replicate and package progeny viruses. Wild-type AAV genomes consists of only two genes: *rep* and *cap*. The *rep* gene encodes four nonstructural Rep proteins involved in the AAV viral life cycle, which mediates the amplification of the *ITR*-flanked genome. The *cap* gene encodes three structural proteins, VP1, VP2, and VP3, which form the icosahedral capsid [21]. The minimum requirement for AAV DNA replication and packaging is the *ITRs*, which flank the AAV genome. Second, HSV is a natural helper virus for AAV packaging. rAAV production relies entirely on the endogenous functions of the AAV Rep and Cap proteins and on the functions of the helper virus. HSV replication-associated proteins can be directly used by AAV for its efficient replication and packaging. The required HSV genes for AAV production include *UL5*, *UL8*, *UL9*, *UL29*, *UL30*, *UL42*, and *UL52*, all of which are components of the HSV core replication system [35, 36].

The Trojan horse-like strategy used in the current study is shown in Fig. 1a. The genomes of the HSV chimeric viruses enter the nucleus following infection of cells. With the assistance of HSV, the Rep replicase specifically recognizes *ITR* cis-elements and replicates the exogenous gene cassette flanked by *ITRs*, resulting in amplification of the copy numbers of the GOI (gene of interest). It can be seen that there are two sources of synergistic accumulation of exogenous gene transcription templates: 1) GOI genes carried by the HSV genome will be amplified with viral genomes replication (Fig.1a①); 2) GOI genes specifically amplified by the AAV replication system, assisted by HSV helper proteins (Fig.1a②), resulting in significant amplification of the number of GOI templates and enhancing protein expression. The H8 virus effectively expressed the AAV Rep protein, and its replication was only slightly slower than that of wildtype HSV, which indicated that Trojan horse-like construction strategy did not affect the replication phenotype of H8 virus. As expected, the green fluorescence of the H8 chimeric virus-infected cells was significantly brighter than that of H1-infected cells. qPCR analysis showed that the *egfp*/viral genome ratio for H8 was significantly higher than that of H1 in both in vitro and in vivo validation experiments (Fig. 1f and Fig. 2b), indicating that the Rep protein expressed by the chimeric vector effectively replicated and amplified the GOI. Similarly, Western blotting showed significantly increased EGFP expression by H8 virus compared with H1.

Previous studies have constructed HSV/AAV chimeric vectors to produce rAAV viruses by HSV-assisted packaging platform or to construct the core plasmids of chimeric HSV amplicon vectors [37, 38]. The difference was that there were some variations in the methods used to construct the chimeric vectors. HSV/AAV amplicon vectors are constructed by combining critical elements from HSV and AAV vectors, which contain only 2 short nucleotide sequences of HSV genome (HSV origin of replication (*oriS*) and HSV DNA cleavage/packaging sequence (*pac*)) and inserting an AAV replication system into the amplicon plasmid backbone, which is then used as a gene delivery tool. The resulting packaged amplicon vectors are structurally HSV-like pseudoviral particles, the genomes of which contain no HSV-1 functional genes. Another application is using hybrid HSV/AAV viruses as a platform for rAAV manufacturing. Unlike the method described in the current study, the production of rAAV particles requires the insertion of the AAV *rep* and *cap* genes together into the HSV genome. However, to enhance transgene expression in this study, only *rep* and cis-acting *ITR* elements need to be inserted into the HSV genome, meaning that AAV particles will not be produced. Here, for the first time, we integrated an AAV replication system directly into the HSV viral genome for the purpose of replicating and amplifying exogenous genes carried by the HSV vector to achieve high-level protein expression. Both in vitro and in vivo experiments demonstrated that the exogenous gene was replicated and amplified, and that there was a significant increase in the expression of the exogenous protein. Furthermore, H8 chimeric viruses possessed the same anterograde transport phenotype as wild-type H129 (Fig. 3 and Additional file 1: Figure S1A), which indicated that the HSV/AAV chimaera construction strategy did not change the infection and anterograde spreading characteristics of H8, and H8 could also be used as an anterograde tracer. Thus, the H8 strain could be more convenient than currently available anterograde tracing tools because it does not require additional immunohistochemical amplification of the fluorescent signal.

C57BL/6 mice infected with H8 showed high levels of EGFP expression at 36 h post-infection, although fewer brain regions were labeled at this point than at 48 h post-infection. However, even at the earlier time point, fluorescence in the labeled regions was sufficiently bright to image. Therefore, the H8 virus provides us with a simpler method to perform time course labeling to dissect neural circuits. Interestingly, the behavior change of mice infected with H8 was milder compared with mice injected with H1 (data not shown). This may be because H8 replicated more slowly than H1 and induced a less powerful inflammatory response. In addition to the merits of the novel H8 virus, there are still some limitations. We recently found that H129-based viral tracers exhibited the phenotype of axon terminal uptake, which led to unintended retrograde labeling of non-specific upstream innervating neurons [31]. H8 may also have the same potential problem, because the Trojan horse-like enhancement strategy described in this study does not change the infection tropism and preference of the H129 virus. On this basis, more research is needed to develop more rigorous HSV anterograde tracing tools.

In summary, we generated a novel HSV anterograde neural circuit tracer, H8, using the Trojan horse-like enhancement strategy. H8 could be used as an anterograde trans-multisynaptic tracer for fast and effective tracing without additional immunostaining. Meanwhile, it was important to caution that H8 might exhibit some non-specific retrograde labeling at longer post-inoculation intervals. To avoid the non-specific tracing, the sampling time should be limited to 3 days or a shorter time post-infection. In addition to being developed as a tracing tool, HSV-based vectors carrying exogenous genes have long been explored for gene therapy applications [39–41]. The unique features of HSV make it particularly attractive for use in transgene delivery to the central nervous system. The Trojan horse-like strategy described here may be applied as a general enhanced expression strategy for HSV-based viral tracers and gene delivery vectors.

## Supplementary information

**Additional file 1: Figure S1.** Labeling efficiency of H8 in anterograde transsynaptic tracing of VTA output neural circuits. (A) Comparison of the labeling performance of H1 and H8 in CNS. Brains infected with H1 or H8 virus were sectioned 72 hpi and serial slices were displayed. Scale bar, 1000 μm. (B) H8 labeled VTA output circuits with a high fluorescence intensity. Ventral tegmental area (VTA), ventral hippocampus (vHIP), ectorhinal cortex (ECT), dorsal raphe nuclei (DR), anterior part of basomedial amygdaloid nucleus (BMA). Scale bar, 100 μm. **Table S1.** The eGFP/β-tubulin protein ratios of H1 or H8 infected cells in vitro. **Table S2.** The eGFP/gD protein ratios of H1 or H8 infected cells in vitro. **Table S3.** The eGFP/β-tubulin protein ratios of H1 or H8 infected mice brains in vivo. **Table S4.** The eGFP/gD protein ratios of H1 or H8 infected mice brains in vivo.

## Data Availability

The datasets used or analysed during the current study are available from the corresponding author on reasonable request.

## Abbreviations

AAV: Adeno-associated virus
AI: Agranular insular cortex
Amy: Amygdala
AON: Anterior olfactory nucleus
BMA: Anterior part of basomedial amygdaloid nucleus
DMEM: Dulbecco’s minimum essential media
DR: Dorsal raphe nuclei
DRN: Dorsal raphe nuclei
ECT: Ectorhinal cortex
EGFP: Enhanced green fluorescent protein
ENT: Entorhinal cortex
GOI: Gene of interest
HIP: Hippocampus
HPI: Hours post infection
HSV: Herpes simplex virus
ITR: Inverted terminal repeat
LHA: Lateral hypothalamus
NAc: Accumbens nucleus
ORB: Orbital cortex
Pir: Piriform cortex
PRV: Pseudorabies virus
PVN: Paraventricular thalamic nucleus
rAAV: Recombinant AAV
SI: Substantia innominate
V1: Primary visual cortex
vHIP: Ventral hippocampus
VTA: Ventral tegmental area
