# Supplementary material for: High-brightness anterograde transneuronal HSV1 H129 tracer modified using a Trojan horse-like strategy
Source: Mol Brain. 2020 Jan 13;13:5. doi: 10.1186/s13041-020-0544-2 (PMC6958791; doi:10.1186/s13041-020-0544-2)
Supplement: Supplementary file 1 — Additional file 1: Figure S1. Labeling efficiency of H8 in anterograde transsynaptic tracing of VTA output neural circuits. (A) Comparison of the labeling performance of H1 and H8 in CNS. Brains infected with H1 or H8 virus were sectioned 72 hpi and serial slices were displayed. Scale bar, 1000 μm. (B) H8 labeled VTA output circuits with a high fluorescence intensity. Ventral tegmental area (VTA), ventral hippocampus (vHIP), ectorhinal cortex (ECT), dorsal raphe nuclei (DR), anterior part of basomedial amygdaloid nucleus (BMA). Scale bar, 100 μm. Table S1. The eGFP/β-tubulin protein ratios of H1 or H8 infected cells in vitro. Table S2. The eGFP/gD protein ratios of H1 or H8 infected cells in vitro. Table S3. The eGFP/β-tubulin protein ratios of H1 or H8 infected mice brains in vivo. Table S4. The eGFP/gD protein ratios of H1 or H8 infected mice brains in vivo. [file 13041_2020_544_MOESM1_ESM.zip › Tabel S2.pdf]

Table S2 The eGFP/gD protein ratios of H1 or H8 infected cells *in vitro*[a]

Sample	gD gray value	gD adjusted gray value	GFP gray value	Adjusted gray scale ratio	Average ratio
H1	23367.71	15959.68	16528.19	1.04	
H1	25166.44	17188.18	17398.57	1.01	1.00 ±0.02
H1	25882.55	17677.27	16831.20	0.95	
H8	14174.78	9681.09	23092.62	2.39	
H8	13151.48	8982.20	23424.75	2.61	2.49 ±0.07[b]
H8	13782.48	9413.16	23191.61	2.46	

[a] BHK cells were infected with H1 or H8 virus at MOI = 0.1 in a six-well plate. After 48 hours, the supernatant was discarded and the cells were rinsed with PBS before scraped down for DNA or protein extraction. Samples were denatured in 5 × loading buffer and separated by using 10% SDS-PAGE electrophoresis. GFP proteins were detected using a rabbit anti-GFP antibody (ab290, Abcam). gD were detected using a monoclonal antibody against gD (ab6507, Abcam). Results were expressed as means ± SEM of each group.

[b] Significant difference ($p<0.001$), compared with H1 group.
